# The Characteristic of Virulence, Biofilm and Antibiotic Resistance of *Klebsiella pneumoniae*

**DOI:** 10.3390/ijerph17176278

**Published:** 2020-08-28

**Authors:** Guoying Wang, Guo Zhao, Xiaoyu Chao, Longxiang Xie, Hongju Wang

**Affiliations:** Institute of Biomedical Informatics, School of Basic Medical Sciences, Henan University, Kaifeng 475004, China; medwgy@163.com (G.W.); zhaoguo006@126.com (G.Z.); a17334815782@126.com (X.C.)

**Keywords:** *K. pneumoniae*, pathogenicity, biofilm, multidrug-resistant

## Abstract

*Klebsiella pneumoniae* is an important gram-negative opportunistic pathogen that causes a variety of infectious diseases, including urinary tract infections, bacteremia, pneumonia, and liver abscesses. With the emergence of multidrug-resistant (MDR) and *hypervirulent K. pneumoniae* (*hvKP*) strains, the rapid spread of these clinical strains in geography is particularly worrying. However, the detailed mechanisms of virulence and antibiotic resistance in *K. pneumoniae* are still not very clear. Therefore, studying and elucidating the pathogenic mechanisms and drug resistance mechanism of *K. pneumoniae* infection are important parts of current medical research. In this paper, we systematically summarized the virulence, biofilm, and antibiotic tolerance mechanisms of *K. pneumoniae*, and explored the application of whole genome sequencing and global proteomics, which will provide new clues for clinical treatment of *K. pneumoniae*.

## 1. Introduction

*Klebsiella pneumoniae* is a class of gram-negative bacterium that is ubiquitously found on the surface of mucosa in animals, or in the environment (such as water, soil, etc.). In humans, *K. pneumoniae* is concentrated in the gastrointestinal tract, and a few in the nasopharynx, through which the bacteria can enter the blood circulation or other tissues, and then cause infection. In the era of pre-antibiotics, *K. pneumoniae* was a vital pathogen of community-acquired pneumonia (CAP), especially in diabetics and alcoholics. In the era of antibiotics that followed, it became a major cause of medical-related infections in hospitals [1], and a risk factor of severe community-acquired infections [2]. In Singapore, mortality rates of *K. pneumoniae* bacteremia ranged from 20–26% [3]. In China, *K. pneumoniae* accounted for 11.9% of isolated pathogens from ventilator-associated pneumonia (VAP) and intensive care unit (ICU)-acquired pneumonia [4]. In addition, carbapenem-resistant Enterobacteriaceae (CRE) caused by *K. pneumoniae* have been reported to account for 73.9% of 664 clinical samples in a multi-center clinical study that covered 25 “AAA” hospitals in 14 provinces of China [5]. There is no doubt that such a high prevalence and mortality rate of *K. pneumoniae* infection caused a great burden on the country’s health system.

*K. pneumoniae* has many accessory genomes of plasmids and chromosome gene loci. According to the accessory genome, *K. pneumoniae* strains are divided into three types: opportunistic, hypervirulent, and multidrug-resistant (MDR) [6]. Recently, most infections of *K. pneumoniae* were caused by the *classic K. pneumoniae* (*cKp*) strains which remained living in the hospitals, and then caused infection in weak patients. The *cKp* strain appears to be different from the *hvKp*. Genetic factors of high virulence phenotype of *hvKp* are present on a large virulent plasmid, and there may be integrative conjugal elements. *hvKp* infection often occurs in multiple sites and subsequently spreads, which makes it more difficult to treat and control. The variant strain *hvKp*, firstly discovered in the Pacific Circle, can lead to community-acquired, aggressive, and metastatic infections in diabetes or normal immune function with liver abscesses, endophthalmitis, meningitis, and septic arthritis in young people in [7,8,9]. Meanwhile, the emergence and spread of MDR strains is an urgent problem to be solved in the field of *K. pneumoniae*. The characteristic of MDR *K. pneumoniae* is closely related to the antibiotic resistance genes (ARGs) encoded by plasmid. Benefitting from plasmids and transferable genetic elements, *K. pneumoniae* continues to accumulate ARGs in the context of improper antibiotic use, resulting in the emergence of an extremely drug-resistant (XDR) strain with “super resistome” [10]. Therefore, further exploration of mechanisms of virulence and drug resistance in *K. pneumoniae* are urgently needed.

## 2. Virulence

In order to accomplish infection, *K. pneumoniae* must conquer mechanical and chemical barriers first, and evade recognition by the humoral immunity and cellular immunity of the host. Upon entry into the host, the invasive microorganisms are recognized by the immune cells via pattern recognition receptors (PRR), and stimulate the body to produce various immune mediators [11]. The mononuclear phagocyte system plays a critical role in the innate immune response, which has the function of phagocytosis and coordinates the immune response through the affection of cytokines and chemokines. Neutrophils are the effector cells firstly presenting to the infection sites. IL-8 and IL-23 are important mediators involved in this process, which induce IL-17 production to promote granulocytopoiesis [12,13]. IL-12 can also increase the level of IL-17 via the increase of gamma interferon. Besides this, other factors involved in the immune response are the production of IL-1β and other pro-inflammatory factors, including IL-6 and tumor necrosis factor-α (TNF-α) [14].

**Figure 1:**
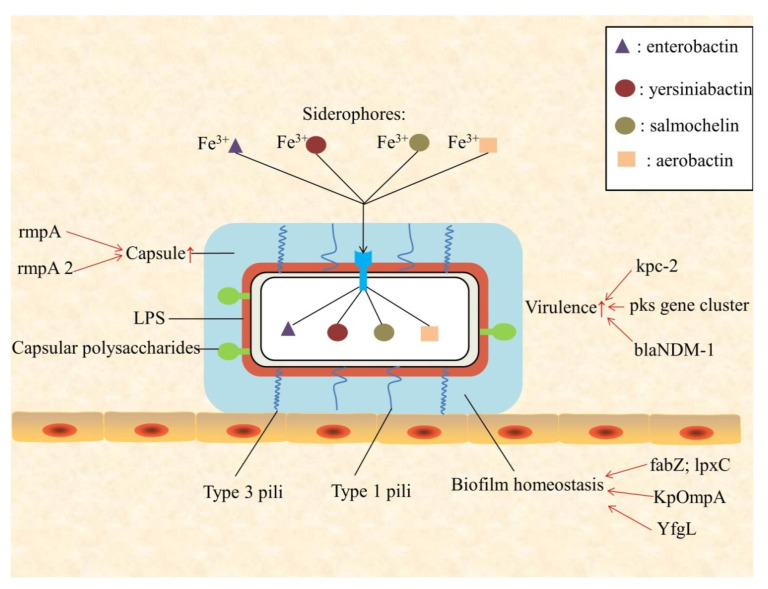
Schematic presentation of *Klebsiella pneumoniae* virulence factors, and biofilm homeostasis.

Currently, there are four virulent factors identified: pili, capsule, lipopolysaccharide (LPS), and iron carriers [15]. *K. pneumoniae* is assembled by adhesins, type 1 and type 3 pili, and it promotes bacterial adhesion to epithelial, immune cells, and abiotic surfaces (Figure 1). *RmpA* is a plasmid-located virulence factor of *K. pneumoniae*, regulating the synthesis of capsular polysaccharides. *RmpA*-carrying strains were significantly associated with high-mucus phenotype of *hvKP*, and with purulent tissue infection such as liver abscess [16]. Although the capsule plays a vital role in protecting *K. pneumoniae* from immune responses of the host, its virulence may be caused by other factors. In fact, LPS in the major *K. pneumoniae* strains may be modified in part, and this leads to *K. pneumoniae* not being recognized by the host cell, while other strains may use the capsule to camouflage LPS to avoid the detection of being toll-like receptor 4 (TLR4) [17,18]. The inflammatory response is inhibited, and the clearance to bacteria is reduced by these modifications. At the same time, the ability to obtain iron is critical to the growth and replication of bacteria. There are four iron-absorbing molecules (iron carriers) in *K. pneumoniae*: enterobactin, yersiniabactin, salmochelin, and aerobactin, respectively. Existing in both typical and highly virulent strains, enteromycin has the highest affinity for iron, and it is considered to be the main iron absorption system. Unlike enteromycin, gastrin and yersinide are more prevalent in *hvKp* than in *cKp* [12,13]. Salmochelin is associated with invasive diseases, and is common in highly virulent *K. pneumoniae* strains that cause severe community-related infections, such as liver abscess and pneumonia [19]. A recent study indicated that *hvKp* strains are capable of producing larger and more activated iron-absorbing molecules, compared with non-virulent strains, which may lead to their virulence and pathogenicity [20].

In addition, some special virulence factors of *K. pneumoniae* strains have been considered in recent years. *Pks* gene clusters can be detected in bloodstream infections (BSIs) caused by *pks* positive (*pks*+) strains. Colibactin is a toxic substance encoded by *pks* gene clusters, which participates in the process of host DNA damage and strains virulence enhancement [21]. By monitoring the prevalence of *K. pneumoniae*-infected bacterial meningitis, Xu et al. found that the high prevalence and mortality of *hvKP* producing *kpc-2* gene meningitis should be paid attention [22]. Some “high risk” sequence types of *K. pneumoniae*, such as STs11, 15, and 383 clones, showed a combination of drug resistance and virulence factor. In these strains, many elements are carried on a large virulent plasmid (such as ST147 with *bla*NDM-1), which usually encodes iron vector genes (such as aerobactin, salmochelin, and enterochelin), heavy metal resistance genes (encoding resistance for tellurite, silver, copper, and lead), and capsule up-regulation genes, *rmpA* and *rmpA2*. The emergence of this combination of resistance and virulence is worrying [23]. It is necessary to conduct in-depth study and epidemic monitoring of some highly virulent strains. Although the clinical features of infection by *hvKp* strains have been fully elucidated, there are only a few reliable biological indicators to distinguish these strains from other *K. pneumoniae* strains. Aerobactin plays an important role in iron carrying, growth, and virulence of *K. pneumoniae*, so it is considered to be the key virulence factor of *hvKP*. The high expression level of aerobactin in strain is a significant feature of the *hvKP* strain. Li et al. revealed that string test combined with aerobactin and *rmpA* index is helpful to improve the detection rate of *hvKP* [16]. We systematically summarized the virulence, biofilm and antibotic resistance related genes, and their functions of *K. pneumoniae* are systematically summarize in Table 1. 

## 3. Biofilm

*K. pneumoniae* can form biofilm, and it accumulates in such cells embedded in the self-generated matrix of the extracellular polymeric substance (Figure 1). Extracellular polymeric substances are complex structures containing polysaccharides, proteins, and DNA. Most clinically apparent *K. pneumoniae* biofilm is formed on the catheter and the inner surface of internal devices [57]. *K. pneumoniae* biofilm can lead to colonization in the respiratory, gastrointestinal, and urinary tracts, as well as the development of invasive infections (especially in immune-deficient patients). The development of *K. pneumoniae* biofilm on the surface of hard objects undergoes the adhesion of cells, the formation of small colonies, maturation, and the propagation as ultimately free-living cells.

The most important surface structures of *K. pneumoniae* involved in the formation process are type 3 pili and capsular polysaccharides (CPs) [58]. Pili mediate steady adhesion, while CPs ultimately affect biofilm structure and intercellular communication. Given the dynamics of biofilm formation and the variability of environmental stimuli, embedded bacterium must have the ability to rapidly and extensively change gene expression. Transcriptional regulation is regulated by quorum sensing, a system coordinating the signals and responses that control gene expression in a microbial population. The putative quorum-sensing-related regulators and autoinducers in *K. pneumoniae* have been identified, but the relevant available data is still incomplete [58]. *K. pneumoniae* with biofilm is protected from the host immune response, in part. This matrix inhibits the proximity of antibodies and antimicrobial peptides, and reduces the effects of complement and phagocytosis [59]. Mutations in some specific genes of *K. pneumoniae* can also affect the function of biofilm. Mostafavi et al. found that *fabZ* and *lpxC* mutations lead to *lpxC* inhibitor-dependent growth of *K. pneumoniae*, which leads to the loss of biofilm homeostasis [24]. In recent years, the research on the outer membrane protein of *K. pneumoniae* has attracted the attention of researchers. Hsieh et al. reported that YfgL (BamB) lipoprotein is involved in the biofilm formation of *K. pneumoniae* and the transcriptional expression of type 1 pili, which is critical for the anti-phagocytosis of *K. pneumoniae* in vivo [25]. Besides this, *K. pneumoniae’s* outer membrane protein A (KpOmpA) is reported to be involved in cell adhesion and cell–cell recognition, as well as immune response of host. Meanwhile, the L3 site on the KpOmpA surface may be related to pathogenicity of *K. pneumoniae* [26]. Oxidative stress may oxidize and destroy the biofilm of *K. pneumoniae*, resulting in the loss of major membrane proteins and activity of *K. pneumoniae* [60]. The above related studies have revealed that *K. pneumoniae’s* biofilm is one of the crucial conditions for maintaining its activity.

## 4. Antibiotic Resistance

### 4.1. Aminoglycoside Resistance Gene

Aminoglycosides, which can inhibit bacteria protein synthesis, were used in antibacterial chemotherapy extensively from 1940 to 1980, until they were replaced by following carbapenems, cephalosporins, and fluoroquinolone drugs [61]. During this time, *K. pneumoniae* acquired antibiotic resistance mechanisms, including drug-modifying enzymes with diverse activities (adenylation, acetylation or phosphorylation). Within ten years, all types of plasmid-mediated resistance genes of *aac*, *aph*, and *ant* gene families in *K. pneumoniae* were found. The reduced use of aminoglycoside slowed the evolution of the novel resistant genes until the 16SrRNA methylase occurred, which was belonged to the *armA* gene family, encoding an enzyme that blocks the binding of aminoglycoside antibiotics to the 16SrRNA [27]. These resistant genes are encoded by plasmids in *K. pneumoniae*, whereas drug-modifying enzymes have narrow-spectrum activity, and 16 rRNA methylase confers resistance to almost all aminoglycosides, including plazomicin and recently developed aminoglycosides [62]. However, an epidemic surveillance, conducted by Cirit et al. in Turkey and Syria in 2019, found that the aminoglycoside resistance of clinically isolated *K. pneumoniae* was mainly mediated by *aac (3)-II*, rather than 16SrRNA methylase [63]. The chromosomal location of *armA* gene family in *K. pneumoniae* has merely been reported once before [64]. Other known plasmids that mediate the activity of 16S rRNA methylases, including the *NpmA* and *Rmt* gene family, have also been identified in *K. pneumoniae* [61], but there is still no evidence of chromosomal localization.

The mechanism of *K. pneumoniae*’s chromosomal tolerance to aminoglycosides includes modification of cell permeability (because of changes in the *KpnEF* efflux pump systems and *AcrAB-TolC*, assuming loss of porins *KpnO*) and aminoglycoside-modifying enzymes (AMEs) genes. Deletion of *AcrAB-TolC* increased the sensitivity of tobramycin and gentamicin [28], whereas the *kpnEF* mutant showed significant resistance to tobramycin and spectinomycin [29], but only slightly tolerated gentamicin and streptomycin. It indicated that the osmotic device has a different affinity for different aminoglycosides. Studies have reported that in vitro *KpnO* pore proteins are directly involved in aminoglycoside resistance, and they can lead to the tolerance of tobramycin, streptomycin, and spectinomycin [30]. No mutations conferring resistance by target modification, such as *rpsL* or *rrs*, have been identified in the *K. pneumoniae* clinical strains. The causes may be high fitness costs and reduced virulence associated with *rpsL* mutations, so they are relatively rare [31]. Among gentamicin- or amikacin-resistant *K. pneumoniae* strains, *acc (6′)-Ib* was considered to be the most widespread, followed by *acc (3′)-II, aph (3′)-IV*, and *ant (3′)-I* [65]. In addition, aminoglycoside resistance was also found in strains producing β-lactamases, which was realized by multiple drug resistance coding genes in plasmids, and *K. pneumoniae* strains coexisting with *rmtC* and *bla*NDM-1 gene were isolated from Turkish clinical samples [66]. Hence, we should not only pay attention to the emergence of aminoglycoside resistance genes, but also pay attention to multidrug-resistant *K. pneumoniae*.

### 4.2. Quinolone Resistance Gene

Quinolone antibiotics target bacteria topoisomerases in order to inhibit DNA replication. These antibiotics have been applied clinically since the 1960s, but they are more widely used after the use of the first class of fluoroquinolones in the 1980s, which leads to the evolution of bacterial fluoroquinolone resistance [67]. The mechanism of *K. pneumoniae* resistance to fluoroquinolone mainly includes mutation of target gene, MDR efflux pump production, and modification of enzymes and target protection proteins [68].

*K. pneumoniae* treated with the first-generation quinolone drug nalidixic acid and the first-generation fluoroquinolone drug ofloxacin was also accompanied by chromosomal resistance of nalidixic acid and ofloxacin. The primary resistance mechanism is the mutation of DNA gyrase (*gyrA-gyrB* subunit) binding to the quinolone on the chromosome and topoisomerase IV (*parC-parE* subunit). Mutations in *parC* and *gyrA* in *K. pneumoniae* were found earlier, and more commonly than those in *gyrB* and *parE* [32,69].

Studies have reported that changes in cell permeability in *K. pneumoniae* are also involved in quinolone resistance, mainly including *OmpK36* deficiency [33], overexpression of the multidrug efflux pump gene *acrAB* [34], and non-alteration of *kdeA* [35]. Induction of these multidrug efflux pump genes into sensitive *E. coli* in vitro will increase their drug resistance. Another important efflux pump from *OqxAB* in *K. pneumoniae*, which was originally thought to derive from the chromosome, has now been shown to be involved in plasmid-mediated quinolone resistance (PMQR), and is widespread in other bacteria [36]. The efflux pump regulator was also revealed to be participated in the quinolone resistance of *K. pneumoniae* [70].

Another class of quinolone resistance genes includes the PMQR determinant, which is present not only in *K. pneumoniae*, but also in other Enterobacteriaceae species. PMQR determinant is considered to be one of the key factors mediating fluoroquinolone resistance in *K. pneumoniae* [71,72]. These genes contain the *qnr* gene, which encodes a protein family that protects DNA gyrase and topoisomerase IV from the inhibition of quinolone. The first *qnr* gene was found on the *K. pneumoniae* plasmid isolated in USA in 1994. *QnrS1*, *qnrD*, *qnrB*, and *oqxAB* pump genes could be detected in *K. pneumoniae* multidrug-resistant strains [73]. Although these proteins are chromosomally encoded in other gram-negative bacteria (including *Shewanella algae*, *Citrobacter* spp, *Stenotrophomonas maltophilia*, and *Serratia marcescens*) [37], there is still no evidence that the *qnr* gene is on the chromosome of *K. pneumoniae*. This suggests that the *qnr* gene may be one of the genes encoding *K. pneumoniae* quinolone-resistant plasmids.

Another PMQR gene, *aa(6′)-Ib-cr*, is considered as the only gene in *K. pneumoniae*, which is responsible for quinolone modification [37]. It can inactivate narrow-spectrum quinolones, such as ciprofloxacin and norfloxacin, which carry the substrate of this enzyme, the unsubstituted piperazine group. Originally it was found to be encoded by the plasmid of *K. pneumoniae*, but recently was also found on chromosomes as well. Aminoglycoside resistance is usually accompanied by β-lactam and quinolone resistance; *aa(6′)-Ib-cr* confers aminoglycosides (kanamycin, tobramycin, and amikacin) and quinolones (norfloxacin and ciprofloxacin) resistance [74].

### 4.3. β-lactam Resistance Gene

β-lactam antibiotics have been used clinically since the 1940s, and are a major class of human medicine-specified antimicrobials. Since then, hundreds of various β-lactamases in intestinal pathogens have evolved, and the number is quite alarming (>2000) and diverse [75].

The penicillin resistance of *K. pneumoniae* was first found in 1960s, which also led to the discovery of the first class of β-lactamase genes (*bla*SHV-1 and *bla*TEM-1). Twenty years later, the first broad-spectrum β-lactamase gene *bla*SHV-2 was identified in *K. pneumoniae* obtained from ICU patients in Germany. This gene exhibits a broad spectrum of β-lactam tolerance activities (cephalosporins and monochosamines included) and is defined as the first discovered ESBL gene. Soon, another plasmid-mediated ESBL variant, *bla*TEM-3, was found in *K. pneumoniae* in France [38]. Since the emergence of ESBLs in *K. pneumoniae* during the period 1990–2000s, it has become the main ESBL-borne pathogen in the outbreak of nosocomial infection. Of the clinical strains of *K. pneumoniae* in all Iraq and Spain hospitals, 40% of the strains are ESBL [76]. During this period, the *K. pneumoniae* strain, which contains major *bla*TEM and *bla*SHV β-lactamases, has a high incidence, and is prevalent in many countries [77]. In 2000, due to the availability of plasmids and transposons encoding *bla*CTX-M type ESBL*s*, the types of ESBL*s* in *K. pneumoniae* caused by iatrogenic outbreaks was altered [39]. Some studies have found that β-lactam resistance can be activated by *ramA*, and the excessive production of *ramA* in *K. pneumoniae* plays an important role in improving acquired β-lactamase-mediated β-lactam resistance. Proteomic analyses show that this enhancement is mainly achieved by activating efflux pump production [40].

Another type of ESBL gene is transferred to *K. pneumoniae* through horizontal gene transfer (HGT), *bla*OXA type ESBLs included [41] and scarce genes *bla*GES and *bla*SFO [42], or *bla*PER, *bla*TLA, and *bla*VEB [43] and *bla*KLUC-5 [44]. In addition, β-lactamase genes that are tolerant to inhibitors have emerged, and they encode enzymes that are partially inhibited by tazobactam and clavulanic acid [75]. In the 30 years since their appearance, the *K. pneumonia* that produced ESBL have increased worldwide. Based on the reports of World Health Organization (WHO), *K. pneumoniae* with ESBL has reached the rate of 50% prevalence in numerous parts of the world, and a resistance rate of 30% in the community, indicating its ubiquity of resistance.

### 4.4. Polymyxin Resistance Gene

Polymyxin disrupts membrane integrity by displacement of extracellular ions (Ca^2+^/Mg^2+^) with binding to negatively-charged lipopolysaccharide (LPS), and causing cell lysis [78]. The history of *K. pneumoniae* polymyxin resistance is shorter compared with other antibiotics, due to the limiting use of medicine in the 1980s and 2000s. Clinically, the first colistin-resistant *Klebsiella aerogenes* (now categorized as *Streptococcus pneumoniae*) was isolated when firstly used [79]. In the early 2000s, as more and more carbapenemase XDR *K. pneumonia* (CPKP) strains appeared, polymyxin became the last class of drugs used [80]. The first nosocomial infection of polymyxin non-sensitive MDR *K. pneumoniae* was found in Greece in 2004 [80]. Since then, there are more and more clinical reports on the emergence of polymyxin-resistant strains.

The main mechanism of *K. pneumoniae* polymyxin resistance is target modification by chromosomal mechanism, also known as “LPS modification system”. Strains with this complex system can change the structure of LPS and cause negative ion reduction by affecting on polymyxin binding [10]. These changes in LPS are due to mutations in core genes responsible for the maturation of lipid A (*lpxM* and its regulator *ramA*) [45,46] and lipid A neutralization, amino arabinose (*pbgP*, *pmrE*), an additional combination of phosphoethanolamine (pmrC), or palmitate (*pagP*) [47,48]. Multiple LPS modified gene regulators, such as *phoPQ*, *pmrA*, and *pmrD*, are also involved in polymyxin resistance. Mutations in one of the two other regulatory genes (resulting in overexpression of *prmB* or inactivation of *mgrB*) are also sufficient to cause polymyxin resistance [49,50]. In 2015, Wright et al. reported that another underlying pathway for LPS modification is *TupA-like*/glycosyltransferase and *CrrAB* regulatory systems [81]. Another mechanism of *K. pneumoniae* colistin resistance is capsular polysaccharide (which may camouflage charged molecules on the outer membrane) [29], as well as high expression of efflux pumps *AcrAB-TolC* and *KpnEF* (due to positive regulation by *RarA*) [46]. Pal et al. found that the deletion of glycosyltransferase *WcaJ* in *K. pneumoniae* made the mutant strain show non-mucus phenotype, which increased polymyxin resistance [51]. Plasmid-mediated polymyxin resistance has merely been reported recently, and the *mcr-1* gene has been identified in China. This gene encodes a family of phosphoethanolamine transferases that share the same activity as PmrC and can bind to phosphoethanolamine [52]. Previous studies have shown that polymyxin resistance occurs in patients, rather than being caused by infection, so limiting the use of polymyxin can reduce *mcr-1*-mediated polymyxin resistance [82]. Phenotypic screening of acquired polymyxin resistance mediated by the *mcr-1* gene includes Ethylene Diamine Tetraacetic Acid (EDTA) and dipicolinic acid (DPA) test [83] and MALDIxin test [84].

### 4.5. Tigecycline Resistance Gene

Tigecycline is the first generation of glycylcycline [85], which has been used since 2005 and is used to treat *K. pneumoniae* infection. Tigecycline is a promising drug with broad-spectrum antibacterial activity, and is effective even for ESBL-derived strains [86]. Shortly after the first usage, a *K. pneumoniae* MDR strain (reduced tigecycline sensitivity, MIC = 4 μg/mL) was isolated in a hospital [87]. The known tigecycline tolerance mechanisms are chromosomally encoded, including modifications of the 30S and 16S ribosomal unit targets, as well as changes in cell permeability. Studies have shown that overexpression of efflux pumps *AcrAB-TolC* and *OqxAB* and changes in the expression levels of their regulators (*RamA*, *RamR, RarA*, and *AcrR*) can also lead to tigecycline resistance [53]. The mutation of *RamR* gene can increase the expression of *RamA* [88]. In addition, *Lon* and *rpsJ* genes were also associated with tigecycline resistance in *K. pneumoniae* [54]. Decreased transcript levels of porin *ompK35K* can also lead to enhanced resistance of *K. pneumoniae* strains [55]. The first mutation in the ribosome protein that causes a decrease in sensitivity occurs on the *rpsJ*-encoded protein S10 [89]. The tetracycline-resistant efflux pumps encoded by the *tetA* gene were found in non-sensitive *K. pneumoniae* isolates, but their mechanism of resistance to tigecycline remains unclear [56].

## 5. Application of Whole Genome Sequencing

Whole genome sequencing enables in-depth identification of bacteria and will provide an effective tool to study and compare in-hospital infections and outbreaks [90] and epidemiological surveillance [91]. To better understand the virulence and prevalence of the *K. pneumoniae* strain, Brisse et al. developed a free BIGSdb-Kp database [92], which can help scientists to quickly obtain medical and epidemiological information from the *K. pneumoniae* genome sequence. In addition, several studies have used high-throughput sequencing to obtain and compare the genomes of endemic high- virulent (CG23) and almost avirulent MDR strains (CG258). Bialek-Davenet et al. had shown that CG258 strain is almost virulence-free but exists various drug resistance-associated genes, such as mutated *gyrA* and *parC* genes in the quinolone resistance-determining region. Meanwhile, most of the genes of MDR and high virulent strains do not overlap [92]. Struve et al. have shown that the CG23 clinical strain is a highly efficient colonizer of the human intestinal tract [93]. They detected large virulence plasmids encoding two iron vectors (bacteriocin and salmochelin) in all *hvKP* strains, and found that these strains also have other iron carriers, such as ICE-related Yersin, colibactin, and microbacteria E492. At present, the application of whole genome sequencing in the field of *K. pneumoniae* also includes exploring the virulence, biofilm formation, and drug resistance mechanism of *K. pneumoniae* at the genome level. Rimoldi et al. found that the existence of *bla*KPC gene is related to carbapenem resistance and the relationship between the type 3 pili of *K. pneumoniae* and iron carrier genes by virulome profile [94]. Meletis et al. revealed that the *NDM-1*-encoding *K. pneumoniae* capsular serotype is determined by the nucleotide sequence of the *wzc* gene. The carbapenem-resistant *K. pneumoniae* genome includes 16 drug resistance genes; 12 located in plasmids and 4 in the chromosome [95]. Founou et al. sequenced the whole genome of ESBL-producing *K. pneumoniae* by Illumina MiSeq platform, and found that ESBL-*K. pneumoniae* contains a variety of β-lactamase genes, including *bla*OXA-1, *bla*TEM-1b, *bla*SHV-1, *bla*CTX-M-15, and other drug resistance genes. The replicon plasmid types of ESBL-*K. pneumoniae* were also detected [96]. Generally, whole genome sequencing has been used in various fields of *K. pneumoniae* study, and we believe that it will become a major tool for *K. pneumoniae* study in the future.

## 6. Application of Global Proteomics

Proteomics has been widely used in major aspects of the bacteria field, such as the identification of bacteria [97] and the development of vaccines [98]. This technique can help us to better analyze the molecular mechanisms of bacterial infection and bacteria-host interaction [99]. Kamaladevi et al. showed that *K. pneumoniae* mainly affected the metabolic pathway of the host during infection, and further experiments showed that *K. pneumoniae* destroyed the host’s defense system by inhibiting the PI3K/AKT/mTOR pathway [100]. Proteomics has been applied to investigate the mechanism of drug resistance in *K. pneumoniae*. Sharma et al. used proteomics (LC-MS/MS) and bioinformatics methods to analyze the possible relationship between *K. pneumoniae* and carbapenem resistance, and found that 52 related overexpressed proteins and their interactive proteins may have contributed to the survival of *K. pneumoniae* under meropenem stress and the emergence of meropenem resistance through diverse mechanisms or multiple pathways [101]. The application of proteomics can also be used as an effective treatment for *K. pneumoniae*. Anand et al. used SDS-PAGE and NLC-MS/MS to find that phage therapy of *K. pneumoniae* through the nasal approach is an effective measure [102]. Therefore, we can believe that more breakthroughs in the field of *K. pneumoniae* will be brought by the application of proteomics.

## 7. Clinical Study of *K. pneumoniae*

To avoid the large-scale dissemination of *K. pneumoniae*, it is necessary to conduct epidemiological surveillance for *K. pneumoniae*, especially extensively antibiotic-resistant strains [103]. Zhang et al. reported a retrospective case-control study of 138 clinical samples with carbapenem-resistant *K. pneumoniae* (CRKP) bloodstream infections (BSI). They concluded that hematological malignancies (odds ratio (OR) = 4.712, (95% CI = 2.181–10.180), *p* < 0.001) and the use of cephalosporin (OR = 3.427, (95% CI = 1.513–7.766), *p* = 0.003) were related with the occurrence of CRKP BSI, while septic shock (OR = 6.418, (95% CI = 1.342–30.686), *p* = 0.020), mechanical ventilation (OR = 9.502, (95% CI = 2.098–43.033), *p* = 0.003) and CRKP infection (OR = 9.171, (95% CI = 1.546–54.416), *p* = 0.015) were independent mortality predictors of *K. pneumoniae* BSI [104]. Consistent with this finding, Demir et al. found that the strongest independent predictors of ESBL-*K. pneumoniae* colonization were mechanical ventilation (OR = 4.28, *p* = 0.000) and hospitalization for longer than 14 days (OR = 6.97, *p* = 0.000) through the research in the pediatric wards [105]. Gastrointestinal colonization is considered the risk factor of *K. pneumoniae* infection in ICU patients [106]. Gorrie et al. conducted a 1-year prospective cohort study based on 498 ICU patients. They revealed that *K. pneumoniae* colonization is an important risk factor for ICU infection (OR = 6.9, *p* < 0.001). About 50% of *K. pneumoniae* infection comes from the patient’s own microbiota [107].

## 8. Conclusions

*K. pneumoniae* has recently become a notorious virulent factor, due to the increase in the number of seriously infected patients. Considering the evolutionary diversity of clinical strains, it is believed that many infection models, including pneumonia, liver abscess, and GI intestinal colonization, as well as different strains, may be better known for these pathogens. Fortunately, increasing studies have been reported to use high-throughput methods to identify virulent factors as a relatively straightforward way to explore innate immune responses and virulent factors. Despite this, we still do not know how *K. pneumoniae* interacts with the different components of the innate immune responses, how its virulent factors overcome host responses and allow *K. pneumoniae* to replicate and construct niches, and how to accelerate the transform of antibiotic resistance mechanism research and clinic treatment strategy. We believe that further study of the biology, physiology, and interactions with host of *K. pneumoniae* will provide important clues to fight *K. pneumoniae* infection.

## Figures and Tables

**Table 1:** The virulence, biofilm and antibotic resistance related genes in *K. pneumoniae*.

The Characteristic of *K. Pneumoniae*	Gene Name	Function	References
Virulence	*rmpA*	Synthesis of capsular polysaccharides; high-mucus phenotype of *hvKP*	[16]
	*rmpA2*	Capsule up-regulation	[23]
	*enterobactin*	Iron carriers; growth and replication of bacteria	[12,13]
	*yersiniabactin*	Iron carriers; growth and replication of bacteria	[12,13]
	*salmochelin*	Iron carriers; growth and replication of bacteria; highly virulent *K. pneumoniae*	[12,13,19]
	*aerobactin*	Iron carriers; growth and replication of bacteria; detection rate of *hvKP*; highly virulent *K. pneumoniae*	[12,13,16]
	*pks gene cluster*	Host DNA damage; strains virulence enhancement	[21]
	*kpc-2*	High prevalence and mortality of *hvKP*	[22]
	*bla*NDM-1	Large virulent plasmid	[23]
Biofilm	*fabZ; lpxC*	Biofilm homeostasis	[24]
	*YfgL (BamB)*	biofilm formation; transcriptional expression of type 1 pili	[25]
	*KpOmpA*	Cell-cell recognition, adhesion, and immune response; pathogenicity	[26]
Aminoglycoside resistance	*16S rRNA methylase*	Encoding an enzyme that blocks the binding of aminoglycoside antibiotics to the 16S rRNA	[27]
	*aac families*	Plasmid-mediated resistance genes	[27]
	*ant families*	Plasmid-mediated resistance genes	[27]
	*aph families*	Plasmid-mediated resistance genes	[27]
	*AcrAB-TolC*	Efflux pump systems; resistance to tobramycin and gentamicin	[28]
	*kpnEF*	Efflux pump systems; significant resistance to tobramycin and spectinomycin	[29]
	*KpnO*	Directly involved in aminoglycoside resistance; resistance of tobramycin, streptomycin and spectinomycin	[30]
	*rrs or rpsL*	*rpsL* mutations associated with high fitness costs and reduced virulence	[31]
Quinolone resistance	*DNA gyrase (gyrA-gyrB subunit) Topoisomerase IV* *(parC-parE subunit)*	Resistance of nalidixic acid and ofloxacin	[32]
	*OmpK36*	Cell permeability	[33]
	*acrAB*	Cell permeability	[34]
	*kdeA*	Cell permeability	[35]
	*OqxAB*	Efflux pump; plasmid-mediated quinolone resistance	[36]
	*qnr*	Encoding a family of proteins that protect DNA gyrase and topoisomerase IV from quinolone inhibitory activity	[37]
	*aa(6′)-Ib-cr*	Quinolone modification	[37]
β-lactam resistance	*bla*SHV-1 and *bla*TEM-1	Penicillin resistance	[38]
	*bla*SHV-2	Extended-spectrum β-lactamase (ESBL) gene	[38]
	*bla*TEM-3	plasmid-mediated ESBL variant	[38]
	*bla*CTX-M	ESBLs in *K. pneumoniae* caused by iatrogenic outbreaks	[39]
	*ramA*	Activating efflux pump; increasing acquired β-lactamase-mediated β-lactam resistance	[40]
	*bla*OXA, *bla*GES, *bla*SFO, *bla*PER, *bla*TLA, *bla*VEB and *bla* KLUC-5	Horizontal gene transfer acquisition	[41,42,43,44]
Polymyxin resistance	*lpxM, ramA*	Maturation of lipid A and lipid A neutralization	[45,46]
	*pbgP, pmrE*	Combination of amino arabinose	[47,48]
	pmrC	Combination of phosphoethanolamine	[47,48]
	*pagP*	Combination of palmitate	[47,48]
	*phoPQ*, *pmrA*, *pmrD* and *mgrB*	LPS modified gene regulators	[49,50]
	*RarA*	High expression of efflux pumps *AcrAB-TolC* and *KpnEF*	[46]
	*WcaJ*	Non-mucus phenotype; increasing polymyxin resistance	[51]
	*mcr-1*	Encoding a family of phosphoethanolamine transferases that can bind to phosphoethanolamine	[52]
Tigecycline resistance	*AcrAB-TolC, OqxAB*	Overexpression of efflux pumps lead to tigecycline resistance	[53]
	*RarA, RamA, RamR* and *AcrR*	Regulators of efflux pumps	[53]
	*Lon* and *rpsJ*	Encoding ribosome protein S10	[54]
	*ompK35K*	Decreased transcript level of porin *ompK35K* can also lead to enhanced resistance	[55]
	*tetA*	Encoding tetracycline-resistant efflux pumps	[56]

## References

[1] Podschun R., Ullmann U. (1998). *Klebsiella* spp. as nosocomial pathogens: Epidemiology, taxonomy, typing methods, and pathogenicity factors. Clin. Microbiol. Rev..

[2] Holt K.E., Wertheim H., Zadoks R.N., Baker S., Whitehouse C.A., Dance D., Jenney A., Connor T.R., Hsu L.Y., Severin J. (2015). Genomic analysis of diversity, population structure, virulence, and antimicrobial resistance in Klebsiella pneumoniae, an urgent threat to public health. Proc. Natl. Acad. Sci. USA.

[3] Chew K.L., Lin R.T.P., Teo J.W.P. (2017). Klebsiella pneumoniae in Singapore: Hypervirulent Infections and the Carbapenemase Threat. Front. Cell. Infect. Microbiol..

[4] Zhang Y., Yao Z., Zhan S., Yang Z., Wei D., Zhang J., Li J., Kyaw M.H. (2014). Disease burden of intensive care unit-acquired pneumonia in China: A systematic review and meta-analysis. Int. J. Infect. Dis..

[5] Zhang Y., Wang Q., Yin Y., Chen H., Jin L., Gu B., Xie L., Yang C., Ma X., Li H. (2018). Epidemiology of Carbapenem-Resistant Enterobacteriaceae Infections: Report from the China CRE Network. Antimicrob. Agents Chemother..

[6] Martin R.M., Bachman M.A. (2018). Colonization, Infection, and the Accessory Genome of Klebsiella pneumoniae. Front. Cell. Infect. Microbiol..

[7] Cubero M., Grau I., Tubau F., Pallares R., Dominguez M.A., Linares J., Ardanuy C. (2016). Hypervirulent Klebsiella pneumoniae clones causing bacteraemia in adults in a teaching hospital in Barcelona, Spain (2007–2013). Clin. Microbiol. Infect..

[8] Kishibe S., Okubo Y., Morino S., Hirotaki S., Tame T., Aoki K., Ishii Y., Ota N., Shimomura S., Sakakibara H. (2016). Pediatric hypervirulent Klebsiella pneumoniae septic arthritis. Pediatr. Int..

[9] Russo T.A., Marr C.M. (2019). Hypervirulent Klebsiella pneumoniae. Clin. Microbiol. Rev..

[10] Navon-Venezia S., Kondratyeva K., Carattoli A. (2017). Klebsiella pneumoniae: A major worldwide source and shuttle for antibiotic resistance. FEMS Microbiol. Rev..

[11] Piperaki E.T., Syrogiannopoulos G.A., Tzouvelekis L.S., Daikos G.L. (2017). Klebsiella pneumoniae: Virulence, Biofilm and Antimicrobial Resistance. Pediatr. Infect. Dis. J..

[12] Paczosa M.K., Mecsas J. (2016). Klebsiella pneumoniae: Going on the Offense with a Strong Defense. Microbiol. Mol. Biol. Rev..

[13] Happel K.I., Dubin P.J., Zheng M., Ghilardi N., Lockhart C., Quinton L.J., Odden A.R., Shellito J.E., Bagby G.J., Nelson S. (2005). Divergent roles of IL-23 and IL-12 in host defense against Klebsiella pneumoniae. J. Exp. Med..

[14] Hua K.F., Yang F.L., Chiu H.W., Chou J.C., Dong W.C., Lin C.N., Lin C.Y., Wang J.T., Li L.H., Chiu H.W. (2015). Capsular Polysaccharide Is Involved in NLRP3 Inflammasome Activation by Klebsiella pneumoniae Serotype K1. Infect. Immun..

[15] Shon A.S., Bajwa R.P., Russo T.A. (2013). Hypervirulent (hypermucoviscous) Klebsiella pneumoniae: A new and dangerous breed. Virulence.

[16] Li G., Sun S., Zhao Z.Y., Sun Y. (2019). The pathogenicity of rmpA or aerobactin-positive Klebsiella pneumoniae in infected mice. J. Int. Med. Res..

[17] Merino S., Camprubi S., Alberti S., Benedi V.J., Tomas J.M. (1992). Mechanisms of Klebsiella pneumoniae resistance to complement-mediated killing. Infect. Immun..

[18] Llobet E., Martinez-Moliner V., Moranta D., Dahlstrom K.M., Regueiro V., Tomas A., Cano V., Perez-Gutierrez C., Frank C.G., Fernandez-Carrasco H. (2015). Deciphering tissue-induced Klebsiella pneumoniae lipid A structure. Proc. Natl. Acad. Sci. USA.

[19] Lam M.M.C., Wyres K.L., Judd L.M., Wick R.R., Jenney A., Brisse S., Holt K.E. (2018). Tracking key virulence loci encoding aerobactin and salmochelin siderophore synthesis in Klebsiella pneumoniae. Genome Med..

[20] Russo T.A., Shon A.S., Beanan J.M., Olson R., MacDonald U., Pomakov A.O., Visitacion M.P. (2011). Hypervirulent *K. pneumoniae* secretes more and more active iron-acquisition molecules than “classical” *K. pneumoniae* thereby enhancing its virulence. PLoS ONE.

[21] Lan Y., Zhou M., Jian Z., Yan Q., Wang S., Liu W. (2019). Prevalence of pks gene cluster and characteristics of Klebsiella pneumoniae-induced bloodstream infections. J. Clin. Lab. Anal..

[22] Xu M., Fu Y., Fang Y., Xu H., Kong H., Liu Y., Chen Y., Li L. (2019). High prevalence of KPC-2-producing hypervirulent Klebsiella pneumoniae causing meningitis in Eastern China. Infect. Drug Resist..

[23] Turton J., Davies F., Turton J., Perry C., Payne Z., Pike R. (2019). Hybrid Resistance and Virulence Plasmids in “High-Risk” Clones of Klebsiella pneumoniae, Including Those Carrying blaNDM-5. Microorganisms.

[24] Mostafavi M., Wang L., Xie L., Takeoka K.T., Richie D.L., Casey F., Ruzin A., Sawyer W.S., Rath C.M., Wei J.R. (2018). Interplay of Klebsiella pneumoniae fabZ and lpxC Mutations Leads to LpxC Inhibitor-Dependent Growth Resulting from Loss of Membrane Homeostasis. mSphere.

[25] Hsieh P.F., Hsu C.R., Chen C.T., Lin T.L., Wang J.T. (2016). The Klebsiella pneumoniae YfgL (BamB) lipoprotein contributes to outer membrane protein biogenesis, type-1 fimbriae expression, anti-phagocytosis, and in vivo virulence. Virulence.

[26] Saurel O., Iordanov I., Nars G., Demange P., Le Marchand T., Andreas L.B., Pintacuda G., Milon A. (2017). Local and Global Dynamics in Klebsiella pneumoniae Outer Membrane Protein a in Lipid Bilayers Probed at Atomic Resolution. J. Am. Chem. Soc..

[27] Doi Y., Wachino J.I., Arakawa Y. (2016). Aminoglycoside Resistance: The Emergence of Acquired 16S Ribosomal RNA Methyltransferases. Infect. Dis. Clin. N. Am..

[28] Peirano G., Ahmed-Bentley J., Fuller J., Rubin J.E., Pitout J.D. (2014). Travel-related carbapenemase-producing Gram-negative bacteria in Alberta, Canada: The first 3 years. J. Clin. Microbiol..

[29] Srinivasan V.B., Rajamohan G. (2013). KpnEF, a new member of the Klebsiella pneumoniae cell envelope stress response regulon, is an SMR-type efflux pump involved in broad-spectrum antimicrobial resistance. Antimicrob. Agents Chemother..

[30] Srinivasan V.B., Venkataramaiah M., Mondal A., Vaidyanathan V., Govil T., Rajamohan G. (2012). Functional characterization of a novel outer membrane porin KpnO, regulated by PhoBR two-component system in Klebsiella pneumoniae NTUH-K2044. PLoS ONE.

[31] Tsai Y.K., Liou C.H., Lin J.C., Ma L., Fung C.P., Chang F.Y., Siu L.K. (2014). A suitable streptomycin-resistant mutant for constructing unmarked in-frame gene deletions using rpsL as a counter-selection marker. PLoS ONE.

[32] Nam Y.S., Cho S.Y., Yang H.Y., Park K.S., Jang J.H., Kim Y.T., Jeong J.W., Suh J.T., Lee H.J. (2013). Investigation of mutation distribution in DNA gyrase and topoisomerase IV genes in ciprofloxacin-non-susceptible Enterobacteriaceae isolated from blood cultures in a tertiary care university hospital in South Korea, 2005–2010. Int. J. Antimicrob. Agents.

[33] Martinez-Martinez L., Hernandez-Alles S., Alberti S., Tomas J.M., Benedi V.J., Jacoby G.A. (1996). In vivo selection of porin-deficient mutants of Klebsiella pneumoniae with increased resistance to cefoxitin and expanded-spectrum-cephalosporins. Antimicrob. Agents Chemother..

[34] Mazzariol A., Zuliani J., Cornaglia G., Rossolini G.M., Fontana R. (2002). AcrAB Efflux System: Expression and Contribution to Fluoroquinolone Resistance in *Klebsiella* spp.. Antimicrob. Agents Chemother..

[35] Ping Y., Ogawa W., Kuroda T., Tsuchiya T. (2007). Gene cloning and characterization of KdeA, a multidrug efflux pump from Klebsiella pneumoniae. Biol. Pharm. Bull..

[36] Wong M.H., Chan E.W., Chen S. (2015). Evolution and dissemination of OqxAB-like efflux pumps, an emerging quinolone resistance determinant among members of Enterobacteriaceae. Antimicrob. Agents Chemother..

[37] Ruiz E., Saenz Y., Zarazaga M., Rocha-Gracia R., Martinez-Martinez L., Arlet G., Torres C. (2012). qnr, aac(6’)-Ib-cr and qepA genes in Escherichia coli and *Klebsiella* spp.: Genetic environments and plasmid and chromosomal location. J. Antimicrob. Chemother..

[38] Sirot D., Sirot J., Labia R., Morand A., Courvalin P., Darfeuille-Michaud A., Perroux R., Cluzel R. (1987). Transferable resistance to third-generation cephalosporins in clinical isolates of Klebsiella pneumoniae: Identification of CTX-1, a novel beta-lactamase. J. Antimicrob. Chemother..

[39] Calbo E., Garau J. (2015). The changing epidemiology of hospital outbreaks due to ESBL-producing Klebsiella pneumoniae: The CTX-M-15 type consolidation. Future Microbiol..

[40] Jimenez-Castellanos J.C., Wan Nur Ismah W.A.K., Takebayashi Y., Findlay J., Schneiders T., Heesom K.J., Avison M.B. (2018). Envelope proteome changes driven by RamA overproduction in Klebsiella pneumoniae that enhance acquired beta-lactam resistance. J. Antimicrob. Chemother..

[41] Evans B.A., Amyes S.G. (2014). OXA beta-lactamases. Clin. Microbiol. Rev..

[42] Bradford P.A. (2001). Extended-spectrum beta-lactamases in the 21st century: Characterization, epidemiology, and detection of this important resistance threat. Clin. Microbiol. Rev..

[43] Philippon A., Slama P., Deny P., Labia R. (2016). A Structure-Based Classification of Class A beta-Lactamases, a Broadly Diverse Family of Enzymes. Clin. Microbiol. Rev..

[44] Li P., Shen K., Zhang Y., Ying J., Zhu T., Liu Y., Xu L., Lin C., Zhang K., Li P. (2018). Characterization of a Novel blaKLUC Variant with Reduced beta-Lactam Resistance From an IncA/C Group Plasmid in a Clinical Klebsiella pneumoniae Isolate. Front. Microbiol..

[45] Clements A., Tull D., Jenney A.W., Farn J.L., Kim S.H., Bishop R.E., McPhee J.B., Hancock R.E., Hartland E.L., Pearse M.J. (2007). Secondary acylation of Klebsiella pneumoniae lipopolysaccharide contributes to sensitivity to antibacterial peptides. J. Biol. Chem..

[46] De Majumdar S., Veleba M., Finn S., Fanning S., Schneiders T. (2013). Elucidating the regulon of multidrug resistance regulator RarA in Klebsiella pneumoniae. Antimicrob. Agents Chemother..

[47] Mitrophanov A.Y., Jewett M.W., Hadley T.J., Groisman E.A. (2008). Evolution and dynamics of regulatory architectures controlling polymyxin B resistance in enteric bacteria. PLoS Genet..

[48] Llobet E., Campos M.A., Gimenez P., Moranta D., Bengoechea J.A. (2011). Analysis of the networks controlling the antimicrobial-peptide-dependent induction of Klebsiella pneumoniae virulence factors. Infect. Immun..

[49] Jayol A., Poirel L., Brink A., Villegas M.V., Yilmaz M., Nordmann P. (2014). Resistance to colistin associated with a single amino acid change in protein PmrB among Klebsiella pneumoniae isolates of worldwide origin. Antimicrob. Agents Chemother..

[50] Poirel L., Jayol A., Bontron S., Villegas M.V., Ozdamar M., Turkoglu S., Nordmann P. (2015). The mgrB gene as a key target for acquired resistance to colistin in Klebsiella pneumoniae. J. Antimicrob. Chemother..

[51] Pal S., Verma J., Mallick S., Rastogi S.K., Kumar A., Ghosh A.S. (2019). Absence of the glycosyltransferase WcaJ in Klebsiella pneumoniae ATCC13883 affects biofilm formation, increases polymyxin resistance and reduces murine macrophage activation. Microbiology.

[52] Liu Y.Y., Wang Y., Walsh T.R., Yi L.X., Zhang R., Spencer J., Doi Y., Tian G., Dong B., Huang X. (2016). Emergence of plasmid-mediated colistin resistance mechanism MCR-1 in animals and human beings in China: A microbiological and molecular biological study. Lancet Infect. Dis..

[53] Osei Sekyere J., Govinden U., Bester L.A., Essack S.Y. (2016). Colistin and tigecycline resistance in carbapenemase-producing Gram-negative bacteria: Emerging resistance mechanisms and detection methods. J. Appl. Microbiol..

[54] Fang L., Chen Q., Shi K., Li X., Shi Q., He F., Zhou J., Yu Y., Hua X. (2016). Step-Wise Increase in Tigecycline Resistance in Klebsiella pneumoniae Associated with Mutations in ramR, lon and rpsJ. PLoS ONE.

[55] Kallman O., Motakefi A., Wretlind B., Kalin M., Olsson-Liljequist B., Giske C.G. (2008). Cefuroxime non-susceptibility in multidrug-resistant Klebsiella pneumoniae overexpressing ramA and acrA and expressing ompK35 at reduced levels. J. Antimicrob. Chemother..

[56] Ahn C., Yoon S.S., Yong T.S., Jeong S.H., Lee K. (2016). The Resistance Mechanism and Clonal Distribution of Tigecycline-Nonsusceptible Klebsiella pneumoniae Isolates in Korea. Yonsei Med. J..

[57] Hall-Stoodley L., Costerton J.W., Stoodley P. (2004). Bacterial biofilms: From the natural environment to infectious diseases. Nat. Rev. Microbiol..

[58] Clegg S., Murphy C.N. (2016). Epidemiology and Virulence of Klebsiella pneumoniae. Microbiol. Spectr..

[59] Fux C.A., Costerton J.W., Stewart P.S., Stoodley P. (2005). Survival strategies of infectious biofilms. Trends Microbiol..

[60] Yang S.K., Yusoff K., Ajat M., Thomas W., Abushelaibi A., Akseer R., Lim S.E., Lai K.S. (2019). Disruption of KPC-producing Klebsiella pneumoniae membrane via induction of oxidative stress by cinnamon bark (*Cinnamomum verum* J. Presl) essential oil. PLoS ONE.

[61] Krause K.M., Serio A.W., Kane T.R., Connolly L.E. (2016). Aminoglycosides: An Overview. Cold Spring Harb. Perspect. Med..

[62] Poulikakos P., Falagas M.E. (2013). Aminoglycoside therapy in infectious diseases. Expert Opin. Pharmacother..

[63] Cirit O.S., Fernandez-Martinez M., Yayla B., Martinez-Martinez L. (2019). Aminoglycoside resistance determinants in multiresistant Escherichia coli and Klebsiella pneumoniae clinical isolates from Turkish and Syrian patients. Acta Microbiol. Immunol. Hung..

[64] Yu F., Wang L., Pan J., Yao D., Chen C., Zhu T., Lou Q., Hu J., Wu Y., Zhang X. (2009). Prevalence of 16S rRNA methylase genes in Klebsiella pneumoniae isolates from a Chinese teaching hospital: Coexistence of rmtB and armA genes in the same isolate. Diagn. Microbiol. Infect. Dis..

[65] El-Badawy M.F., Tawakol W.M., El-Far S.W., Maghrabi I.A., Al-Ghamdi S.A., Mansy M.S., Ashour M.S., Shohayeb M.M. (2017). Molecular Identification of Aminoglycoside-Modifying Enzymes and Plasmid-Mediated Quinolone Resistance Genes among Klebsiella pneumoniae Clinical Isolates Recovered from Egyptian Patients. Int. J. Microbiol..

[66] Guven Gokmen T., Nagiyev T., Meral M., Onlen C., Heydari F., Koksal F. (2016). NDM-1 and rmtC-Producing Klebsiella pneumoniae Isolates in Turkey. Jundishapur. J. Microbiol..

[67] Naeem A., Badshah S.L., Muska M., Ahmad N., Khan K. (2016). The Current Case of Quinolones: Synthetic Approaches and Antibacterial Activity. Molecules.

[68] Redgrave L.S., Sutton S.B., Webber M.A., Piddock L.J. (2014). Fluoroquinolone resistance: Mechanisms, impact on bacteria, and role in evolutionary success. Trends Microbiol..

[69] Guillard T., de Jong A., Limelette A., Lebreil A.L., Madoux J., de Champs C., ComPath Study G. (2016). Characterization of quinolone resistance mechanisms in Enterobacteriaceae recovered from diseased companion animals in Europe. Vet. Microbiol..

[70] Zheng J.X., Lin Z.W., Sun X., Lin W.H., Chen Z., Wu Y., Qi G.B., Deng Q.W., Qu D., Yu Z.J. (2018). Overexpression of OqxAB and MacAB efflux pumps contributes to eravacycline resistance and heteroresistance in clinical isolates of Klebsiella pneumoniae. Emerg. Microbes Infect..

[71] Jacoby G.A., Strahilevitz J., Hooper D.C. (2014). Plasmid-mediated quinolone resistance. Microbiol. Spectr..

[72] Mirzaii M., Jamshidi S., Zamanzadeh M., Marashifard M., Malek Hosseini S.A.A., Haeili M., Jahanbin F., Mansouri F., Darban-Sarokhalil D., Khoramrooz S.S. (2018). Determination of gyrA and parC mutations and prevalence of plasmid-mediated quinolone resistance genes in Escherichia coli and Klebsiella pneumoniae isolated from patients with urinary tract infection in Iran. J. Glob. Antimicrob. Resist..

[73] Surleac M., Czobor Barbu I., Paraschiv S., Popa L.I., Gheorghe I., Marutescu L., Popa M., Sarbu I., Talapan D., Nita M. (2020). Whole genome sequencing snapshot of multi-drug resistant Klebsiella pneumoniae strains from hospitals and receiving wastewater treatment plants in Southern Romania. PLoS ONE.

[74] Schultsz C., Geerlings S. (2012). Plasmid-mediated resistance in Enterobacteriaceae: Changing landscape and implications for therapy. Drugs.

[75] Bush K. (2010). Bench-to-bedside review: The role of beta-lactamases in antibiotic-resistant Gram-negative infections. Crit. Care.

[76] Canton R., Akova M., Carmeli Y., Giske C.G., Glupczynski Y., Gniadkowski M., Livermore D.M., Miriagou V., Naas T., Rossolini G.M. (2012). Rapid evolution and spread of carbapenemases among Enterobacteriaceae in Europe. Clin. Microbiol. Infect..

[77] Livermore D.M. (2012). Current epidemiology and growing resistance of gram-negative pathogens. Korean J. Intern. Med..

[78] Falagas M.E., Kasiakou S.K. (2005). Colistin: The revival of polymyxins for the management of multidrug-resistant gram-negative bacterial infections. Clin. Infect. Dis..

[79] Davis B., Lilly H.A., Lowbury E.J. (1969). Gram-negative bacilli in burns. J. Clin. Pathol..

[80] Antoniadou A., Kontopidou F., Poulakou G., Koratzanis E., Galani I., Papadomichelakis E., Kopterides P., Souli M., Armaganidis A., Giamarellou H. (2007). Colistin-resistant isolates of Klebsiella pneumoniae emerging in intensive care unit patients: First report of a multiclonal cluster. J. Antimicrob. Chemother..

[81] Wright M.S., Suzuki Y., Jones M.B., Marshall S.H., Rudin S.D., van Duin D., Kaye K., Jacobs M.R., Bonomo R.A., Adams M.D. (2015). Genomic and transcriptomic analyses of colistin-resistant clinical isolates of Klebsiella pneumoniae reveal multiple pathways of resistance. Antimicrob. Agents Chemother..

[82] Macesic N., Nelson B., McConville T.H., Giddins M.J., Green D.A., Stump S., Gomez-Simmonds A., Annavajhala M.K., Uhlemann A.C. (2020). Emergence of Polymyxin Resistance in Clinical Klebsiella pneumoniae Through Diverse Genetic Adaptations: A Genomic, Retrospective Cohort Study. Clin. Infect. Dis..

[83] Wink P.L., Caierao J., Nunes A.G.A., Collar G.D.S., Martins J.B., Dalmolin T.V., Pilonetto M., Barth A.L. (2020). Evaluation of EDTA and Dipicolinic Acid in Broth Microdilution with Polymyxin B as a Phenotypic Test to Detect the mcr-1 Gene. Microb. Drug Resist..

[84] Dortet L., Broda A., Bernabeu S., Glupczynski Y., Bogaerts P., Bonnin R., Naas T., Filloux A., Larrouy-Maumus G. (2020). Optimization of the MALDIxin test for the rapid identification of colistin resistance in Klebsiella pneumoniae using MALDI-TOF MS. J. Antimicrob. Chemother..

[85] Livermore D.M. (2005). Tigecycline: What is it, and where should it be used?. J. Antimicrob. Chemother..

[86] Golan Y. (2015). Empiric therapy for hospital-acquired, Gram-negative complicated intra-abdominal infection and complicated urinary tract infections: A systematic literature review of current and emerging treatment options. BMC Infect. Dis..

[87] Ruzin A., Visalli M.A., Keeney D., Bradford P.A. (2005). Influence of transcriptional activator RamA on expression of multidrug efflux pump AcrAB and tigecycline susceptibility in Klebsiella pneumoniae. Antimicrob. Agents Chemother..

[88] Li R., Han Y., Zhou Y., Du Z., Wu H., Wang J., Chen Y. (2017). Tigecycline Susceptibility and Molecular Resistance Mechanisms among Clinical Klebsiella pneumoniae Strains Isolated during Non-Tigecycline Treatment. Microb. Drug Resist..

[89] Villa L., Feudi C., Fortini D., Garcia-Fernandez A., Carattoli A. (2014). Genomics of KPC-producing Klebsiella pneumoniae sequence type 512 clone highlights the role of RamR and ribosomal S10 protein mutations in conferring tigecycline resistance. Antimicrob. Agents chemother..

[90] Wang X., Xie Y., Li G., Liu J., Li X., Tian L., Sun J., Ou H.Y., Qu H. (2018). Whole-Genome-Sequencing characterization of bloodstream infection-causing hypervirulent Klebsiella pneumoniae of capsular serotype K2 and ST374. Virulence.

[91] Lepuschitz S., Schill S., Stoeger A., Pekard-Amenitsch S., Huhulescu S., Inreiter N., Hartl R., Kerschner H., Sorschag S., Springer B. (2019). Whole genome sequencing reveals resemblance between ESBL-producing and carbapenem resistant Klebsiella pneumoniae isolates from Austrian rivers and clinical isolates from hospitals. Sci. Total Environ..

[92] Bialek-Davenet S., Criscuolo A., Ailloud F., Passet V., Jones L., Delannoy-Vieillard A.S., Garin B., Le Hello S., Arlet G., Nicolas-Chanoine M.H. (2014). Genomic definition of hypervirulent and multidrug-resistant Klebsiella pneumoniae clonal groups. Emerg. Infect. Dis..

[93] Struve C., Roe C.C., Stegger M., Stahlhut S.G., Hansen D.S., Engelthaler D.M., Andersen P.S., Driebe E.M., Keim P., Krogfelt K.A. (2015). Mapping the Evolution of Hypervirulent Klebsiella pneumoniae. mBio.

[94] Rimoldi S.G., Gentile B., Pagani C., Di Gregorio A., Anselmo A., Palozzi A.M., Fortunato A., Pittiglio V., Ridolfo A.L., Gismondo M.R. (2017). Whole genome sequencing for the molecular characterization of carbapenem-resistant Klebsiella pneumoniae strains isolated at the Italian ASST Fatebenefratelli Sacco Hospital, 2012-2014. BMC Infect. Dis..

[95] Meletis G., Chatzopoulou F., Chatzidimitriou D., Tsingerlioti F., Botziori C., Tzimagiorgis G., Skoura L. (2019). Whole Genome Sequencing of NDM-1-Producing ST11 Klebsiella pneumoniae Isolated in a Private Laboratory in Greece. Microb. Drug Resist..

[96] Founou R.C., Founou L.L., Allam M., Ismail A., Essack S.Y. (2019). Whole Genome Sequencing of Extended Spectrum beta-lactamase (ESBL)-producing Klebsiella pneumoniae Isolated from Hospitalized Patients in KwaZulu-Natal, South Africa. Sci. Rep..

[97] Boulund F., Karlsson R., Gonzales-Siles L., Johnning A., Karami N., Al-Bayati O., Ahren C., Moore E.R.B., Kristiansson E. (2017). Typing and Characterization of Bacteria Using Bottom-up Tandem Mass Spectrometry Proteomics. Mol. Cell. Proteom..

[98] Bittaye M., Cash P. (2015). Streptococcus pneumoniae proteomics: Determinants of pathogenesis and vaccine development. Expert Rev. Proteom..

[99] Saleh S., Staes A., Deborggraeve S., Gevaert K. (2019). Targeted Proteomics for Studying Pathogenic Bacteria. Proteomics.

[100] Kamaladevi A., Balamurugan K. (2017). Global Proteomics Revealed Klebsiella pneumoniae Induced Autophagy and Oxidative Stress in Caenorhabditis elegans by Inhibiting PI3K/AKT/mTOR Pathway during Infection. Front. Cell. Infect. Microbiol..

[101] Sharma D., Garg A., Kumar M., Khan A.U. (2019). Proteome profiling of carbapenem-resistant *K. pneumoniae* clinical isolate (NDM-4): Exploring the mechanism of resistance and potential drug targets. J. Proteom..

[102] Anand T., Virmani N., Kumar S., Mohanty A.K., Pavulraj S., Bera B.C., Vaid R.K., Ahlawat U., Tripathi B.N. (2019). Phage therapy for treatment of virulent Klebsiella pneumoniae infection in a mouse model. J. Glob. Antimicrob. Resist..

[103] Lee C.R., Lee J.H., Park K.S., Jeon J.H., Kim Y.B., Cha C.J., Jeong B.C., Lee S.H. (2017). Antimicrobial Resistance of Hypervirulent Klebsiella pneumoniae: Epidemiology, Hypervirulence-Associated Determinants, and Resistance Mechanisms. Front. Cell. Infect. Microbiol..

[104] Zhang Y., Guo L.Y., Song W.Q., Wang Y., Dong F., Liu G. (2018). Risk factors for carbapenem-resistant *K. pneumoniae* bloodstream infection and predictors of mortality in Chinese paediatric patients. BMC Infect. Dis..

[105] Demir S., Soysal A., Bakir M., Kaufmann M.E., Yagci A. (2008). Extended-spectrum beta-lactamase-producing Klebsiella pneumoniae in paediatric wards: A nested case-control study. J. Paediatr. Child Health.

[106] Oğuz Mızrakçı S., Arda B., Erdem H.A., Uyar M., Tünger A., Sipahi O.R., Ulusoy S. (2013). [Risk factors for gastrointestinal colonization by ESBL-producing Klebsiella pneumoniae and Escherichia coli in anaesthesiology and reanimation intensive care unit]. Mikrobiyol. Bul..

[107] Gorrie C.L., Mirceta M., Wick R.R., Edwards D.J., Thomson N.R., Strugnell R.A., Pratt N.F., Garlick J.S., Watson K.M., Pilcher D.V. (2017). Gastrointestinal Carriage Is a Major Reservoir of Klebsiella pneumoniae Infection in Intensive Care Patients. Clin. Infect. Dis..

